# Survey of Home‐Use UV Disinfection Products^†^

**DOI:** 10.1111/php.13423

**Published:** 2021-05-04

**Authors:** Marina Khazova, Liam Johnstone, Dzhordzhio Naldzhiev, John B. O'Hagan

**Affiliations:** ^1^ Public Health England Chilton, Didcot UK; ^2^ The Department for Business, Energy and Industrial Strategy Office for Product Safety and Standards Westminster, London UK

## Abstract

The COVID‐19 pandemic provided a commercial opportunity for traders marketing a range of ultraviolet (UV) radiation products for home‐use disinfection. Due to concerns about the efficacy of such products and the potential for harmful levels of UV exposure to people, a range of products were purchased from on‐line trading platforms. Spectral irradiance measurements were carried out to determine whether the products could be effective against the SARS‐CoV‐2 virus and whether they were likely to exceed internationally agreed exposure limits. It was concluded that many of the devices were not effective and many of those that were potentially effective presented a risk to users.

## Graphical abstract

48 devices available for UK consumers in April–July 2020 and marketed for home use UV‐C disinfection were assessed. They included devices for area exposure, handheld wands, enclosures/boxes and one vacuum cleaner; nine out of 48 devices did not emit radiation effective for inactivation of viruses. Effectiveness of disinfection depends on design, surface area covered, direct line‐of‐sight, exposure time and distance between the UV‐C device and the treated surface. Where access to UV radiation was not prevented by in‐built safety features, exposure to emission from these devices may result in a risk to the eyes and skin.

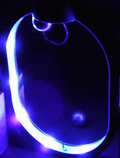

## INTRODUCTION

The acute respiratory syndrome coronavirus 2 (SARS‐CoV‐2) is responsible for more than 116 million confirmed COVID‐19 cases and 2.589 million deaths as recorded on 8 March 2021 (1), as well as a deep drop in global consumption of goods and services and millions of job losses. As the pandemic has progressed, there has been an acceleration in the search for effective controls to limit the spread of the virus.

The use of ultraviolet (UV) radiation may be an important environmental intervention which can reduce both contact spread and airborne transmission of pathogens (2, 3). Solar UV is the primary natural viricidal agent (4, 5, 6) which may help reduce viral load outdoors. However, seasonal changes in Europe with diminishing contribution of sun exposure to viral control (7, 8) and people moving indoors for occupational, educational and most of their recreational activities, has shifted the emphasis to the deployment of affordable measures to abate COVID‐19 transmission including the use of artificial UV radiation in public places and at home.

Ultraviolet radiation, UV‐C in particular, has been used for disinfection of air (9, 10, 11), water (12), surfaces (13, 14) and food for decades (15, 16, 17). The World Health Organization (WHO) recognized it as a means for tuberculosis infection prevention and control (3).

Ultraviolet radiation is generally considered to be carcinogenic, with UV‐B and UV‐A parts of the spectrum being known carcinogens (18). Although there is no evidence that UV‐C alone causes cancer in humans, it can cause erythema, trigger photokeratitis and some UV‐C sources can also emit UV‐B and UV‐A radiation (19, 20).

The likely routes of transmission of the SARS‐CoV‐2 virus may include transfer from the hands following contact with a contaminated surface to the eyes, nose or mouth and through breathing in virions present in droplets or aerosols. Very early in the pandemic, an increasing range of home‐use products became available on on‐line trading platforms. These products were marketed with claims of reducing the risk of catching COVID‐19 and other diseases. However, the rapid proliferation of UV‐C disinfection technology outside the professional sector raised concerns that some devices may pose a risk to human health and/or produce insufficient inactivation of the virus. As a response, the World Health Organization (WHO) and the Commission Internationale de l´Éclairage (CIE) released position statements concerning the use of UV‐C disinfection products and warned against the use of UV disinfection lamps on hands or any other area of skin (2, 21) unless clinically justified. If UV‐C devices are used for disinfecting surfaces or air, in addition to assessment of germicidal potential, personal safety should be evaluated to ensure that recommendations on the limits of exposure specified in the International Commission on Non‐Ionizing Radiation (ICNIRP) guidelines (22) are not exceeded. For conclusive risk assessment and management, appropriate UV measurements are essential.

Early in the pandemic, there was a report of three members of a household in Hong Kong who were hospitalized after using a UV‐C source to disinfect their home. The device was purchased over the internet, and the safety information to the user supplied with the device was not actioned (23). It was reasonably foreseeable that similar incidents would occur elsewhere. Photokeratitis was recorded in seven people in Helsinki, and three of which were exposed to lamps at home, three at their workplace and one at a dentist office (24). The patients reported that they did not follow manufacturer instructions and were directly exposed without skin or eye protection for periods from 10 min to 4 h (24).

In April–July 2020, Public Health England (PHE) together with the Office for Product Safety and Standards (OPSS) carried out a pilot survey of the photobiological safety and potential for viral disinfection of home‐use UV disinfection devices available at that time on the UK on‐line consumer market; 48 devices in total were assessed. The results of this study are reported here.

## MATERIALS AND METHODS

The spectral irradiance was measured at the distance recommended for use in any user information and, where appropriate, at 200 mm required for the classification in accordance with BS EN 62471: 2008 “Photobiological Safety of Lamps and Lamp Systems” (25). Measurements were carried out under environmentally controlled laboratory conditions using a double‐grating IDR300 spectroradiometer (Bentham Instruments, Reading, UK) calibrated using a 1000 W tungsten‐halogen lamp, calibrated for spectral irradiance to the Physikalisch‐Technische Bundensanstalt (PTB, Germany) traceable reference standards (250–800 nm) and Deuterium lamps, calibrated for spectral irradiance to the National Physical Laboratory (NPL, UK) traceable reference standards (200–400 nm).

The initial results suggested two areas of concern: that the products may not be effective for inactivating SARS‐CoV‐2 and/or they may present a risk to the eyes or the skin. Spectral irradiance data were used to calculate:

- *E*_UV_, UV‐effective irradiance weighted with the S(λ) hazard weighting function (actinic hazard) and time t_UV_ to reach the exposure limit of 30 J m^−2^ of the ICNIRP guidelines;
- *E*_VI_, irradiance weighted with the virus inactivation efficacy as a function of wavelength and time to reach 90% inactivation (*t*_VI_^90^) and 99% inactivation (*t*_VI_^99^). The viral inactivation action spectrum was determined from Lytle and Sagripanti (4). The weighted radiant exposure required for 90% inactivation of SARS‐CoV‐2 is taken to be 6.9 and 28 J m^−2^ for 99% inactivation according to Sagripanti and Lytle (5). At the time of writing, there were no internationally agreed weighted exposure values for the inactivation of SARS‐CoV‐2 for real‐world exposure situations, and the values taken from Sagripanti and Lytle (2020) were considered reasonable. However, the data in this paper can be scaled for radiant exposure values from other studies.

To assess the benefit to risk potential, the ratio of times for 90% or 99% viral inactivation with respect to the safe ocular and skin exposure limits was derived as follows:

(1) $$A_{90}=\frac{t_{\text{VI}}^{90}}{t_{\text{IIV}}},$$

(2) $$A_{99}=\frac{t_{\text{VI}}^{99}}{t_{\text{IIV}}}.$$

These values are unique for the device, depend only on the spectral power distribution of emission and could be considered as modified hazard ratios widely used in the assessment of optical radiation safety (26). If *A*_X_ < 1, the required level of inactivation X could be achieved without over‐exposure of the eyes or skin. If *A*_X_ ≥ 1, the required level of inactivation X may result in a risk to the eyes and skin when human access to the radiation is not prevented during use. It should be stressed that *A* < 1 does not mean, at all, that this device is eye‐ and skin‐safe: only that the time for viral inactivation is shorter than the time of ocular‐safe exposure.

## RESULTS AND DISCUSSION

The devices fell into four broad categories: handheld wands (18 units), area exposure units (17 units), enclosures/bags (12 units) and one handheld vacuum cleaner. A total of 24 devices were equipped with mercury lamps and 24 units comprised light‐emitting diodes (LEDs); details are shown in Table 1.

**Table 1:** Samples of home‐use UV disinfection products.

Emitter type/number of units	Area exposure devices	Handheld wands	Enclosure/bags	Vacuum cleaner
Mercury (Hg)	11	8	4	1
UV‐C LED	4	4	7	–
Non‐UV‐C LED	2	3	1	–
Plastic cover over emitters	–	3	–	–

The 24 devices incorporating mercury lamps emitting UV‐C radiation, including the 253.7 nm line, were generally capable of inactivating viruses; with A_90_ in the range of 0.11–0.12 and A_99_ in the range of 0.46–0.47, i.e., 90% inactivation could be achieved in one‐tenth of the time needed to pose a risk to the eyes or skin. However, all the units incorporating mercury lamps were capable of exposing people to levels of UV‐C that could result in erythema or photokeratitis unless access to radiation was prevented by in‐built safety features.

The devices incorporating LEDs comprised exposed UV‐C LEDs, UV‐C LEDs covered with a plastic shield and LEDs emitting in the UV‐A or visible spectral regions. Only devices with exposed UV‐C LED emitters could potentially be useful for viral inactivation; 11 (23%) units either had a plastic cover, which blocked short wavelengths UV, or they emitted UV‐A or visible light only, making these devices unsuitable for disinfection despite claims on the packaging and information to the user. This includes two E27 (medium Edison screw) fitting lamps, one white and one blue, marketed for UV‐C sterilization (*sic*). Note that the term “sterilization” was used on the packaging or the information to the user accompanying many of the products. Sterilization is usually used where the quantity of virus is reduced by at least a factor of one million. This is misleading because none of the 11 products assessed could achieve this level of viral inactivation.

For the devices with LED UV‐C emitters, A_90_ varied in the range of 0.25–0.33 and A_99_ within the range of 1.0–1.35, depending on LED peak wavelength emission. While 90% viral inactivation may be feasible without simultaneous risk to the eyes or skin, 99% eye‐safe inactivation at the same distance is unlikely. Furthermore, the irradiance produced by the LED devices was significantly lower than that emitted by the mercury lamps and the irradiated area was also smaller. Both of the tested area exposure devices with UV‐C LEDs were at least an order of magnitude less effective for inactivation at the same distance compared with devices equipped with mercury lamps. Unlike mercury lamps emitting in 360°, unless fitted with a back reflector, LEDs emit in the forward direction only and may be better suited for irradiating particular surfaces rather than space.

**Figure 1:**
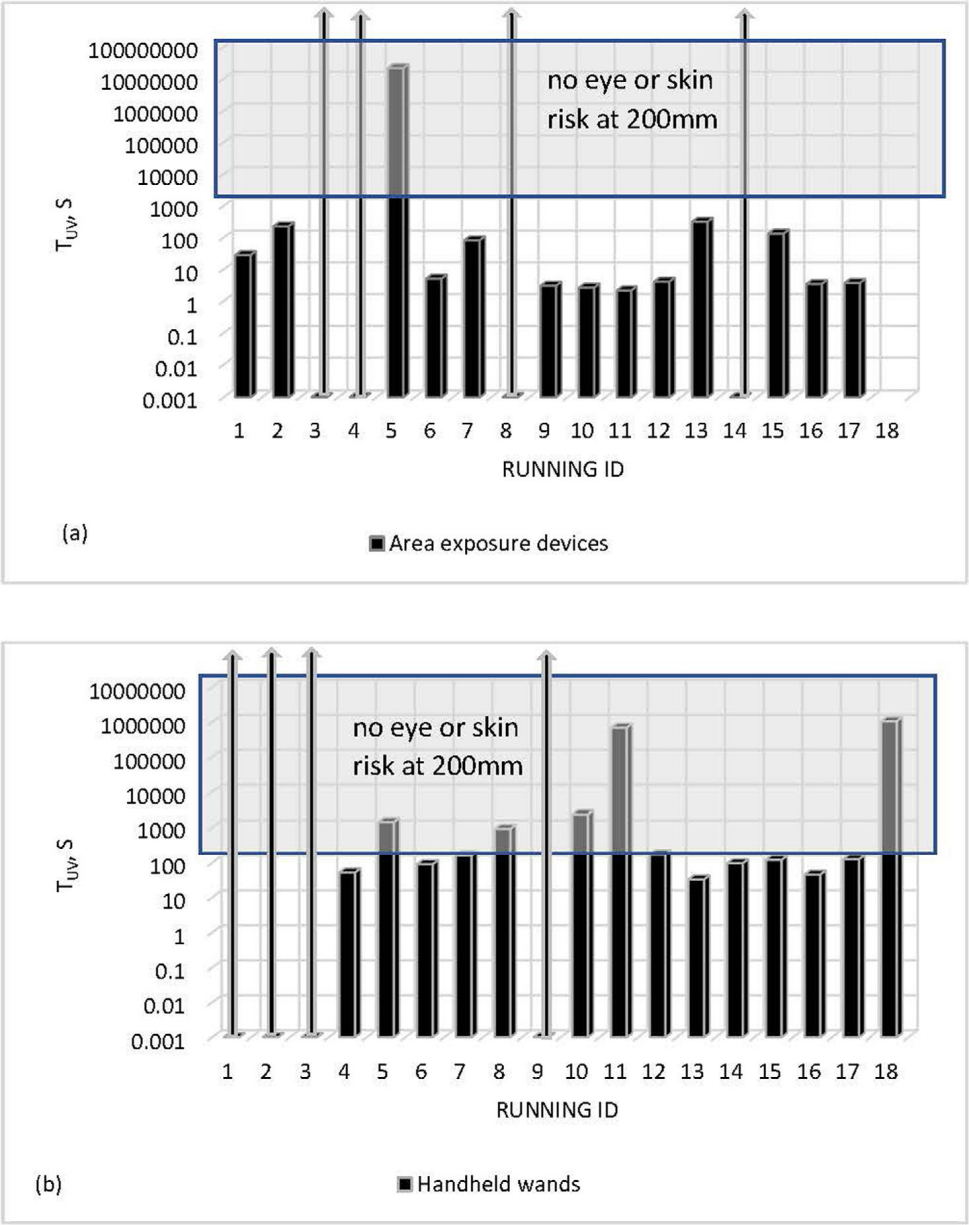
Shortest time to reach the exposure limit of 30 J m^‐^^2^ (*t*_UV_) of area exposure (a) and handheld devices (b) assessed at 200 mm.

Shortest time to reach the exposure limit of 30 J m^‐2^ (*t*_UV_) at arm’s length, taken as 200 mm, for the area exposure devices and the handheld wands is shown in Fig. 1 on a logarithmic scale. Where there was no actinic risk to the eyes from the product at this distance, the data cell is shown as bold arrow.

*t*_UV_ time at a foreseeable accessible distance of 200 mm is, generally, much shorter for area exposure devices than for handheld wands. The shortest time to reach the exposure limit of 30 J m^−2^ of the ICNIRP guidelines for the tested area devices (Fig. 1a) is 2 s and 33 s for the handheld ones (Fig. 1b). For seven out of seventeen tested devices, *t*_UV_ is shorter than 5 s even at 200 mm; four area exposure devices did not emit hazardous actinic radiation, and the emission of one unit was extremely low. Closer to the source, risk of over‐exposure increased, and many area exposure devices presented foreseeable risk to the eyes and skin even at much longer distances. *t*_UV_ was shorter than 1 min for three out of eighteen handheld devices at 200 mm, within the range of 1–3 min for six units, longer than 10 min for five of them; four handheld wands did not emit hazardous actinic radiation.

More than half of the enclosures/bags were equipped with safety features preventing direct access to optical radiation (see Table 2), and ocular exposure was prevented in seven out of twelve devices; one unit did not emit hazardous actinic radiation. The shortest ocular‐safe exposures at 200 mm looking inside working devices were 6 s in one unit and an order of minutes in another one; times were much longer with the others and risk to the eyes could be considered insignificant for those devices. However, three enclosures/boxes had a gravity or tilt sensor as a safety feature preventing direct ocular exposure, but it was possible to place them directly on skin and the time to reach the exposure limit of 30 J m^−2^ of the ICNIRP guidelines could be as short as 10 s.

**Table 2:** Safety features of the UV‐C disinfection devices.

Safety features/number of units	Area exposure devices	Handheld wands	Enclosure/bags	Vacuum cleaner
Motion sensor	1 (of 17)	–	–	–
Gravity sensor or tilt switch	–	5 (of 18)	3 (of 12)	1 (of 1)
Interlock	–	–	4 (of 12)	1 (of 1)

**Figure 2:**
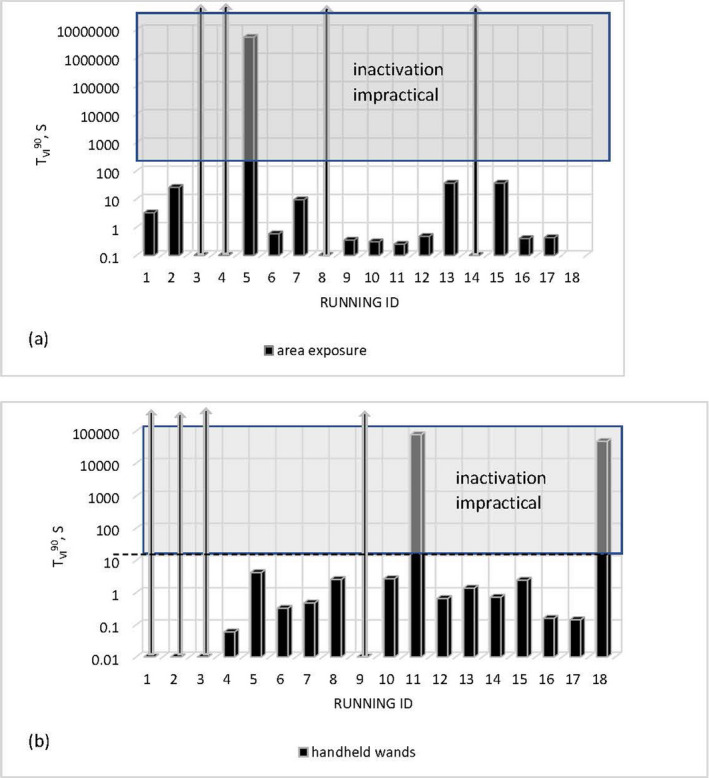
Time required for 90% viral inactivation *t*_VI_^90^ for area exposure (a) devices assessed at treatment distance of 200 mm and handheld wands (b) assessed at treatment distance of 20 mm.

Time required for 90% viral inactivation *t*_VI_^90^ for handheld and area exposure devices is shown in Fig. 2. Where viral inactivation by the product was unlikely, the data cell is shown as bold arrow.

Seven out of seventeen area exposure devices (Fig. 2a) were capable of 90% viral inactivation in less than 1s within the 200 mm distance, two in less than 10s, and three smaller units in less than 1 min; five devices either did not emit UV‐C or UV‐B radiation or the emission level was negligible.

Potential for viral inactivation of handheld wands was assessed at recommended treatment distance of 20 mm. Within 20 mm, seven out of eighteen handheld wands (Fig. 2b) had the potential for viral inactivation in less than 1 s, five units in 1–5 s and six required hours of treatment for 90% even at very close distances. However, a main challenge with the use of UV‐C for area disinfection is shadowing, which means that surfaces not in direct line‐of‐sight of the source may not receive sufficient radiant exposure for effective disinfection.

Accurate quantitative assessment of the potential effectiveness for viral inactivation of enclosures/boxes was not possible due to a combination of fitted safety features, which terminated emission when open or tilted, and geometric factors, for example range of distances from the emitters. Therefore, only an indication of the potential for viral inactivation was made in this case; emission of one unit did not contain UV‐C or UV‐B radiation and viral inactivation was not feasible by this product. It also should be emphasized that items placed inside for disinfection were generally only irradiated on one or two sides, with the other surfaces shielded from the UV by the item placed inside. The effectiveness of all these devices for virus inactivation is critically dependent on line‐of‐sight exposure conditions; the information to the user did not address this.

For the most of tested devices (but not all), user information, and in some cases—direct labelling on the body of device itself, contained warning to avoid direct exposure of the eyes and skin. In addition, a number of home‐use UV‐C disinfection devices incorporated safety measures including gravity and motion sensors, and interlocks, as listed in Table 2.

A motion sensor was fitted in a single area exposure device, effectively preventing people’s presence in the treatment area; all other area exposure devices did not incorporate any safety features and, for the units equipped with mercury lamps, at close distances of 20–50 cm hazardous exposure levels could be reached in seconds. It should be also noted that 3 of the tested units produced ozone within minutes of operation at sufficient levels to require thorough ventilation of the premises. Ozone is a very strong oxidant that may cause irritation to the airways when breathed in and to the eyes; at higher concentrations (27, 28), it may be toxic or interact with materials.

The vacuum cleaner was equipped with pressure and gravity (tilt switch) sensors which disabled UV emission if the cleaning head was tilted or lifted off the treated surface normally preventing human access to hazardous emission. However, it would be possible to operate the device if it was placed on a hand.

Five of the handheld wands incorporated gravity or tilt switch sensors terminating emission if the device was tilted and preventing accidental eye exposure; however, this does not stop accidental or intentional exposure of the skin if, for example, the hands were placed under the unit. Although the risk to the eyes was reduced for such devices, it is reasonably foreseeable that a child could be looking up into a device being used by an adult. Two handheld wands were additionally supplied with protective eyewear.

Most of the enclosures/bags were effective at minimizing the risk of eye exposure by design restrictions; more than 50% (7 out of 12) were additionally equipped with interlocks terminating emission when the device was open or tilted; for those without interlocks, hands could be intentionally placed inside but this risk is small.

Data from the OPSS COVID‐19 Consumer Survey (29) demonstrated that, out of 200 people who purchased UV devices, 31% reported purchasing it for use on their skin and 17% for use on their pets. It can only be hypothesized as to whether this was either a replacement for, or complimentary to, hand washing and the use of hand sanitizer. A purchaser of a portable UV‐C device was considered likely to find a way to expose at least their hands to the UV‐C, even if control measures were incorporated into the product. This was particularly relevant for the wand devices. Based on the consumer survey results, it could be hypothesized that some consumers are unlikely to read warnings. In situations where operating instructions are not followed or are missing, the risk of exposure to UV‐C has led to consumers experiencing photokeratitis or requiring hospitalization (23, 24).

50% of the devices assessed contained low‐pressure mercury lamps. The vulnerability of these lamps to accidental damage varied, but the lamps in several devices appeared very exposed. If the lamp was broken, there would be risks associated with broken glass, plus the chemical hazard associated with mercury.

## CONCLUSION

A total of 48 devices available for UK consumers in April‐July 2020 and marketed for home use‐UV‐C disinfection or sterilization were assessed. Not all devices were marketed specifically for use against SARS‐CoV‐2, but all claimed effectiveness against viruses and bacteria. The surveyed sample set included devices for area exposure, handheld wands, enclosures/boxes and one vacuum cleaner. Devices were equipped with either mercury lamps, UV‐C LEDs or non‐UV‐C LEDs; nine out of 48 devices did not emit radiation effective for inactivation of viruses. OPSS instigated the appropriate corrective actions in relation to the noncompliant and unsafe products identified through testing (30).

Where UV‐C disinfection devices are used as a mitigation measure for preventing viral spread in indoor environments, it is recommended that their efficacy and safety be demonstrated with relevant data. Effectiveness of disinfection depends on multiple parameters including the underlying technology, design of the device, surface area covered, whether surfaces are in direct line‐of‐sight, exposure time and distance between the UV‐C device and the treated surface.

In general, for mercury lamp‐based devices, 90% inactivation could be achieved in one‐tenth of the time needed to pose a risk to the eyes or skin. However, all the units incorporating mercury lamps were capable of exposing people to levels of UV‐C that could result in erythema or photokeratitis unless access to radiation was prevented by in‐built safety features.

Inappropriate use of UV‐C equipment, such as direct exposure of eyes or skin, has been associated with potential health risks. Portable devices should be used with care following manufacturer guidelines, and safety controls should put in place in order to minimize unintended consequences.
